# Dietary Strategies for Maintenance of Clinical Remission in Inflammatory Bowel Diseases: Are We There Yet?

**DOI:** 10.3390/nu12072018

**Published:** 2020-07-07

**Authors:** Konstantinos Gkikas, Konstantinos Gerasimidis, Simon Milling, Umer Z. Ijaz, Richard Hansen, Richard K. Russell

**Affiliations:** 1 Human Nutrition, School of Medicine, Dentistry and Nursing, University of Glasgow, Glasgow Royal Infirmary, Glasgow G31 2ER, UK; k.gkikas.1@research.gla.ac.uk (K.G.); Konstantinos.Gerasimidis@glasgow.ac.uk (K.G.); 2 Institute for Infection, Immunity and Inflammation, University of Glasgow, Glasgow G12 8QQ, UK; Simon.Milling@glasgow.ac.uk; 3 Civil Engineering, School of Engineering, University of Glasgow, Glasgow G12 8QQ, UK; Umer.Ijaz@glasgow.ac.uk; 4 Department of Paediatric Gastroenterology, Royal Hospital for Children, Glasgow G51 4TF, UK; richard.hansen@nhs.net; 5 Department of Paediatric Gastroenterology, Royal Hospital for Sick Children, Edinburgh EH9 1LF, UK

**Keywords:** inflammatory bowel disease, Crohn’s disease, Ulcerative colitis, dietary therapy, maintenance enteral nutrition, dietary triggers, clinical relapse

## Abstract

The etiopathogenesis of Inflammatory bowel disease (IBD) is a result of a complex interaction between host immune response, the gut microbiome and environmental factors, such as diet. Although scientific advances, with the use of biological medications, have revolutionized IBD treatment, the challenge for maintaining clinical remission and delaying clinical relapse is still present. As exclusive enteral nutrition has become a well-established treatment for the induction of remission in pediatric Crohn’s disease, the scientific interest regarding diet in IBD is now focused on the development of follow-on dietary strategies, which aim to suppress colonic inflammation and delay a disease flare. The objective of this review is to present an extensive overview of the dietary strategies, which have been used in the literature to maintain clinical remission in both Crohn’s disease and Ulcerative colitis, and the evidence surrounding the association of dietary components with clinical relapse. We also aim to provide study-related recommendations to be encompassed in future research studies aiming to investigate the role of diet during remission periods in IBD.

## 1. Introduction

Inflammatory bowel diseases (IBD) are chronic, relapsing and debilitating inflammatory disorders, of which Crohn’s disease (CD) and Ulcerative colitis (UC) are the most common [1]. While UC involves only superficial inflammation in the colon and rectum, CD can cause transmural, granulomatous inflammation throughout the gastrointestinal tract, anywhere from mouth to anus. Indicative IBD symptoms include diarrhea, abdominal pain, blood in stool, extra-intestinal manifestations, such as arthritis and delays in growth and puberty in children. IBD prevalence is particularly high in North America, central and northern Europe, including Scotland [2,3]. The rising IBD incidence in newly-industrialized countries adopting a Western type lifestyle [4], serves as a prime example of the significant contribution of environmental factors and especially diet to IBD etiopathogenesis.

Although the complete etiology of IBD remains to be fully understood, the prevailing paradigm suggests a multifaceted interaction between an aberrant immune response and environmental stimuli, mediated through the gut microbiome, in genetically susceptible people. Patients with CD have a distinct, “dysbiotic” gut microbiome profile, compared to healthy people, characterized by lower gut bacterial α diversity, and perhaps lower relative abundance of Faecalibacterium prausnitzii and increased abundance of Escherichia coli [5,6]. The microbial composition differences which have been observed between patients with CD and UC, potentially suggest that the observed microbial signature is at least partially disease-specific [7]. So far, there is inconclusive and contradictory evidence on whether changes in disease activity precede or follow shifts in the gut microbiome [5,8,9,10].

Several epidemiological studies suggest that a Mediterranean-type dietary pattern, comprising mainly fiber-rich sources, such as fruit and vegetables, and ω-3 fatty acid-rich food sources, is associated with reduced risk of IBD development. Conversely, a Western-style diet, consisting mainly of processed food, food additives, red meat and animal fat has been associated with increased risk of IBD onset [11,12,13,14]. However, recent cohort studies have challenged these associations, thus highlighting that epidemiological evidence, surrounding pre-illness dietary intake, and the onset of IBD, is inconclusive [15,16].

The only well-established, evidence-based dietary therapy, used in CD, is exclusive enteral nutrition (EEN). EEN consists of the exclusive consumption of a proprietary formula, typically for 6–8 weeks, and is the first-line treatment for induction of remission in mild and moderate pediatric CD across Europe, Canada and Oceania [17,18]. Multiple lines of research, including systematic reviews, meta-analyses and Cochrane reviews, have demonstrated the efficacy of EEN, particularly in children with CD, with clinical remission rates at least comparable to those from corticosteroids [19,20,21,22]. While, EEN efficacy has not yet been demonstrated consistently in adult populations, it is suggested that this is partly a result of lower compliance, potentially derived from limited formula palatability and a lack of established support mechanisms from the relevant clinical teams [23]. The role of EEN in UC has not yet been extensively studied, and conclusions cannot be drawn regarding its efficacy [24].

Importantly, EEN is able to achieve higher intestinal mucosal healing rates than corticosteroids, in children with CD [25,26]. As mucosal healing is assessed through invasive endoscopic procedures, which are not always practical or ethically acceptable for research purposes, with a couple of notable exceptions [26,27], various proxies have, thus, been used to assess the levels of intestinal inflammation. Fecal calprotectin (FC), a neutrophil-derived protein, has emerged as a non-invasive predictor of mucosal healing and is now established in the clinical setting. EEN has been shown to reduce FC levels in pediatric populations by approximately 50% [28]. EEN, along with other forms of nutritional support, such as total parenteral nutrition (TPN) have also been efficacious in reducing post-operative occurrence in patients with CD undergoing surgery [29,30].

In addition to understanding the induction of remission, it is equally important to identify how remission can be maintained by manipulating the diet. We recently demonstrated a rapid rise in FC levels during the first 2–3 weeks of food reintroduction in children with EEN-induced remission [31]. Hence, the reintroduction of dietary constituents, when patients return to normal diet, is associated with a rise in FC and with reactivation of intestinal inflammation. As patients frequently seek ways to modify their diet to control their disease [32,33], identification of the putative causative dietary components is arguably one of the most important priorities in CD research at present.

One of the main aims of both medical and nutritional treatments is to maintain clinical remission, by suppressing immune response, thereby delaying a disease flare [34]. The most common maintenance therapy in CD is the use of immunosuppressants, such as thiopurines or methotrexate. However, the use of thiopurines has been associated with increased risk of adverse events (e.g., infection, pancreatitis and rarely malignancy) [35,36,37]. Biologics are also increasingly used in up to 50% of children with CD and while clearly efficacious, they are associated with loss of response over time, a well-established side effect profile and high financial burden [38,39,40]. Additionally, relapse rates and the need for surgery in IBD continue to be considerably high, thus, highlighting a therapeutic gap where novel, non-medical therapies, such as nutritional therapies are needed.

The aim of this review was to provide a comprehensive overview of the evidence surrounding dietary strategies for maintenance of remission in IBD, to explore potential dietary triggers of relapse, and to conclude whether current evidence is sufficient to make dietary recommendations for use in clinical practice. Furthermore, we aimed to provide a list with essential and desirable study-related characteristics, which we believe, should form the basis of future research investigating the role of diet in prolonging clinical remission in IBD.

## 2. Literature Review

An extensive literature search was undertaken on PubMed (inception to May 2020), including Medical Subject Heading terms (“inflammatory bowel disease” OR IBD OR Crohn OR colitis) AND (diet OR diet* OR nutrition OR nutr* OR food OR “enteral nutrition” OR polymeric OR elemental). 18,924 records were identified. Eligible studies included intervention and cohort studies investigating the role of diet for the maintenance of clinical remission in both adult and pediatric IBD populations in remission. Animal, in vitro and studies not in English were excluded. We also excluded studies assessing dietary strategies for the induction of remission or perioperative nutritional support, single-nutrient supplement studies, and observational studies associating pre-illness diet with the onset of IBD. After full-text review of 226 articles, 39 studies fulfilled the eligibility criteria. In addition, five studies were identified after cross-checking reference lists from the included papers, subsequently resulting in a final inclusion of 44 studies. Comprehensive evidence tables presenting the included studies and relevant information are displayed in Supplementary file 1.

We first present studies examining the efficacy of food reintroduction protocols in patients with CD, after induction of remission using nutritional therapies. Subsequently, we focus on studies employing enteral nutrition as a maintenance regime in patients with CD, and on studies using ordinary food-based diets for maintenance of clinical remission in both CD and UC. Finally, we present the current evidence on prospective, observational studies exploring the associations with dietary components and risk of relapse in CD and UC.

### 2.1. Food Reintroduction Protocols in CD

Six intervention trials (three randomized controlled trials (RCT) and three non-randomized), and two retrospective studies assessing food reintroduction protocols in 422 patients with CD were included. Six studies recruited only adults [41,42,43,44,45,46], one recruited only children [47] and another had a mixed population [48]. EEN was the most common induction treatment with some studies also using TPN and exclusion diets [41,42,44]. Table 1 presents a summary of the food reintroduction protocols used in studies which included a control group.

#### 2.1.1. Symptom-Alleviating Exclusion Diets

Gradual, single-food reintroduction with exclusion of symptom-aggravating foods was the most commonly used food reintroduction protocol. Jones et al. [41] demonstrated in an RCT that adherence to a symptom-alleviating, food reintroduction diet, resulted in 70% lower clinical relapse rates, assessed by disease activity indices, compared to patients following an unrestricted, fiber-rich diet (intervention: 30% vs. control: 100%, *p* < 0.05). Erythrocyte sedimentation rate (ESR) levels significantly decreased in patients following the exclusion diet, whereas there was no change in the control group. Subsequently, the same research group demonstrated in an uncontrolled trial that patients adhering to exclusion diets had 36% clinical relapse rates after a 12-month follow-up [42]. Riordan et al. [46] showed that patients following a symptom-guided exclusion diet after EEN-induced remission, had marginally, but significantly lower 2-year clinical relapse rates than patients who were administered tapering corticosteroids doses and followed general dietary advice (62% versus 79%, *p* = 0.048).

The efficacy of symptom-guided exclusion diets, following EEN, was assessed in two uncontrolled trials, which used double-blind food challenge tests [43,48]. No difference in clinical relapse rates were observed between patients who followed exclusion diets, compared to those who followed unrestricted diets in the first trial [43]. Similarly, duration of remission did not significantly differ between patients with positive food challenges against those who did not identify symptom-triggering foods in the second trial [48]. Some of the most frequently reported symptom-triggering foods in the above studies were wheat, dairy products, eggs, and yeast-containing products.

#### 2.1.2. Immunoglobulin G Exclusion Diet

Delayed onset of symptoms after food challenges, and the presence of subclinical inflammation in asymptomatic patients, are two disadvantages of relying on symptom-guided elimination diets. Wang et al. [45] aimed to address these limitations by using Immunoglobulin G (IgG) exclusion diets, since high IgG and particularly IgG4 levels might be associated with an abnormal immune reaction to those foods [49]. However, current guidelines suggest that increased IgG4 concentration against food stimuli is a physiological immune reaction representing chronic exposure of the host to specific foods, rather than a food intolerance [50]. Patients in the intervention group excluded foods causing moderate or strong immunoreactivity, based on IgG levels, after testing against 14 commonly consumed food items [45]. No significant differences in relapse rates and endoscopic scores between the intervention and the control group (unrestricted diet) were observed.

#### 2.1.3. LOFFLEX Diet

Woolner et al. [44] tested the efficacy of a low-fat, low-fiber diet (LOFFLEX) in a non-randomized trial, following induction of remission with EEN or TPN. The diet was initially followed for 2–4 weeks and was followed by gradual reintroduction of non-symptom triggering foods. Patients in the control group followed a standard symptom-alleviating, exclusion diet. No significant differences in remission rates were observed between the two groups (LOFFLEX: 44% versus standard: 45%, *p* = not significant [NS]).

#### 2.1.4. Rapid Food Reintroduction Diet

Faiman et al. [47] investigated the efficacy of a rapid food reintroduction regime in a pediatric, retrospective study. Patients in the rapid reintroduction arm followed a low-residue diet for three days and were subsequently allowed to consume an unrestricted diet. There were no significant differences in clinical relapse rates between patients in the rapid arm compared to those following a standard, gradual food reintroduction protocol (rapid: 50% vs. standard: 47%, *p* = NS).

##### Summary

Among the food reintroduction protocols presented here, only exclusion diets which aimed to prevent exacerbation of clinical disease activity and symptoms demonstrated some clinical efficacy. However, one challenge with interpreting previous studies from an era when non-invasive biomarkers of luminal inflammation, such as FC, were unavailable, and endoscopic assessment was not used, is whether gut inflammation parallels symptomatic response. A symptomatic response does not always match with improvements in gut inflammation and the same often applies with alterations in blood inflammatory markers (e.g., C-reactive protein [CRP], ESR). Therefore, the efficacy of these diets in controlling colonic inflammation needs to be demonstrated before they can be recommended as food reintroduction protocols.

### 2.2. Dietary Therapies for Maintenance of Clinical Remission in IBD

#### 2.2.1. Maintenance Enteral Nutrition in CD

Maintenance enteral nutrition (MEN) describes the use of EN formula, that comprises a percentage of the total calorific intake, for maintenance of clinical remission. Nineteen studies assessing the use of MEN in CD were included (Supplementary file 1). Eight were intervention trials (four RCT, four non-randomized) and 11 were cohort studies. Twelve studies recruited adults (*n* = 1076) and seven recruited children (*n* = 392). A comparator group was included in 17/19 (89%) of the studies. Out of the 16 studies which compared clinical relapse rates between patients using MEN and those that did not, 9/16 (56%) of the studies demonstrated lower relapse rates in patients using MEN; 8/11 (73%) in adults [51,52,53,54,55,56,57,58] and 1/5 (20%) in children [59]. Conversely, in 3/11 (27%) adult and 3/5 (60%) pediatric studies, clinical relapse rates did not differ between patients using MEN against those in the comparator group [31,60,61,62,63,64]. In one pediatric study, MEN use led to lower relapse rates only in patients not using any concomitant medication, with no significant differences observed in relapse rates between patients using MEN and azathioprine than those only using azathioprine [65].

**Figure 1:**
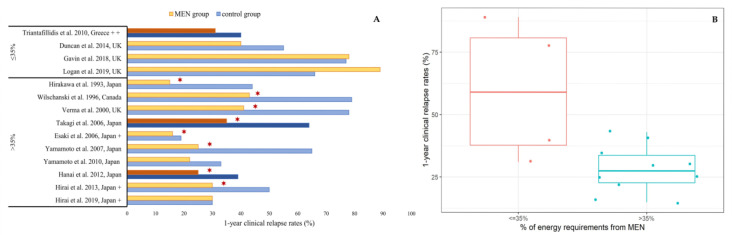
(**A**) Comparison of 1-year clinical relapse rates in patients using MEN against those who did not use MEN in studies reporting the amount of MEN formula consumed (≤35%/>35% of energy requirements). **✶** indicates statistically significant differences in clinical relapse rates between MEN group and control group in the total duration of each study. Dark colored bar charts indicate RCTs. ^+^ Clinical relapse rates in Esaki et al., 2006, Hirai et al., 2013 and Hirai et al., 2019 are approximate numbers extracted from figures. ^++^ only 6-month relapse rates were available. (**B**) Median clinical relapse rates in patients consuming MEN, based on amount of formula consumed (≤35%/>35% of energy requirements).

Out of the 14 studies which had a comparator group and reported the amount of MEN used, 10/14 (71%) used MEN covering >35% of patients’ daily energy requirements and 4/14 (29%) used MEN covering ≤35% (Figure 1). Consumption of >35% MEN was associated with significantly lower relapse rates compared to the control group, in 8/10 (80%) studies. Conversely, in all four studies using ≤35% MEN, there was no additional benefit of MEN use in reducing clinical relapse. Median (Q1, Q3) 1-year clinical relapse rates were 28% (21, 37) amongst patients consuming >35% MEN and 59% (33, 86) in patients consuming ≤35% MEN (Figure 1). In a retrospective, pediatric study, which did not report relapse rates, patients consuming 50% MEN had similar duration of clinical remission compared to those who did not consume MEN [63].

Fifteen studies reported concomitant maintenance medication, with two studies stratifying patients in two different groups depending on the use of MEN with/without additional medication [58,65]. The use of MEN was associated with significantly lower clinical relapse rates, compared to the absence of MEN, in all three studies where aminosalicylates were the main maintenance treatment [53,56,66] and in 2/3 (33%) studies where MEN was used as the sole maintenance treatment [58,60,65]. Use of MEN as an adjunctive therapy to steroids and/or immunosuppressants, was effective in reducing relapse rates in 4/8 (50%) studies [54,58,59,67]. In 3/4 studies in which MEN was not effective in augmenting the efficacy of steroids or immunosuppressants, it was prescribed in low amounts (≤35% of energy intake). Lastly, when infliximab was used as the main maintenance therapy, patients consuming MEN had significantly lower relapse rates, compared to those not using MEN, in 1/3 studies (33%) [57]. However, a recent meta-analysis, which examined the effect of MEN as adjunctive treatment to anti-TNF therapy in patients with both, active and inactive CD, showed that patients using MEN had increased probability of achieving remission than those not using MEN (pooled OR (95%CI, 2.23 [1.60–3.10]) [68].

The type of MEN formula does not seem to affect clinical efficacy, as no significant differences in clinical remission rates were observed between patients using polymeric compared to those using elemental formulas, in an RCT of 33 adults (polymeric: 43% vs. elemental: 42%, *p* = NS) [67].

The use of MEN was associated with further clinical benefits: improved endoscopic indices, reduced mucosal cytokine levels [55], reduction of FC during the early food reintroduction period [31] and increased height [59] and weight z-scores [63] in children. The use of MEN, concomitantly with a low-fat diet, has also been associated with a reduced risk of post-operative disease recurrence, in patients undergoing surgical resection, compared to patients on a normal diet [69]. Interestingly, in two pediatric studies, not included in the present review due to inclusion of patients with active disease, intermittent use of EEN over one year, was associated with a significant reduction in annual steroid use and increased growth velocity [70,71]. This EN regime could serve as an alternative in patients who struggle to consume EN over a large period of time but would eventually require repeated compliance to additional EEN courses in patients with EEN-induced remission.

##### Summary

It could be assumed that the main barrier for the wider use of MEN is low compliance, due to low formula palatability and patients’ fatigue. Compliance was not assessed in studies in which MEN was not efficacious [60,63,64]. Hence, treatment failure could have been a result of low acceptability rather than limited MEN efficacy. Although an intake covering ≤35% of patients’ daily energy requirements is not sufficient to exert beneficial outcomes on the disease trajectory, larger MEN intakes demonstrated significant efficacy in maintaining clinical remission (Figure 1). Considering that immunosuppressants are known to have a significant adverse event profile, MEN could potentially be a safer effective alternative in some patients [72].

#### 2.2.2. Food-Based Therapies

Twelve trials (*n* = 1180) assessed the efficacy of food-based interventions for maintenance of clinical remission [73,74,75,76,77,78,79,80,81,82,83,84,85]. Six included only patients with CD (five in adults, one in children) and six only patients with UC (five in adults, one in children). Table 1 presents an overview of those dietary strategies.

##### CD Studies

Low-Refined, Low-Carbohydrate Diets

One RCT investigated the efficacy of a low-refined carbohydrate diet and another focused on reducing total carbohydrate intake [73,74] as a means of maintaining clinical remission in patients with CD. In the first RCT, 59% of patients adhering to a low-refined carbohydrate, fiber-rich diet relapsed within two years, compared to 64% of patients following a low-fiber diet (*p* = NS) [73]. In the second RCT, adherence to a low-carbohydrate diet was not significantly associated with higher remission rates, compared to a normal diet [74].

Semi-Vegetarian Diet

Chiba et al. [75] investigated the efficacy of a plant-based, semi-vegetarian diet for maintenance of clinical remission in a non-randomized trial, in patients with CD, with medically (infliximab) or surgically induced remission. Details of the diet are presented in Table 1. Patients not adhering to the allowed/disallowed foods were assigned to an omnivorous diet group. Strikingly, all patients in the intervention group maintained clinical remission after a one-year follow-up, with only 1/16 (16%) patients relapsing after two years, compared to 4/6 (67%) patients in the omnivorous diet group (*p* < 0.001). Relapse was assessed as appearance of symptoms requiring medical care. In a subsequent single-group trial, the same research group showed that patients with UC following the same dietary protocol had 39% clinical relapse rates after five years [85].

Anti-IBD Diet

Mutlu et al. [76] assessed the effect of an “anti-IBD” diet, a diet low in animal fat, grains, additives, and high in monounsaturated and ω-3 fatty acids, in a double-blind RCT in patients with CD, only available as an abstract. None of the patients in the “anti-IBD” diet group relapsed after one year, compared to 6/19 (32%) patients receiving a fructooligosaccharide supplement along with normal diet (*p* < 0.05), and to 4/19 (21%) patients receiving placebo along with normal diet (*p* = NS). Furthermore, a significant increase in the abundance of bacteria from the genus Roseburia was observed in the “anti-IBD” diet group.

Low Red and Processed Meat Diet

Albenberg et al. [77] assessed the effect of red and processed meat consumption on relapse risk in patients with CD, in a well-designed RCT. Patients in the intervention group were allowed consumption of no more than one serving of red and processed meat per month, and those in the control group were instructed to consume at least two portions per week. Clinical relapse rates and FC concentration did not significantly differ between the two groups, after a follow-up of 49 weeks.

Crohn’s Disease Exclusion Diet

In a recent RCT, Levine et al. [78] assessed the efficacy of the CD exclusion diet (CDED) with partial enteral nutrition for induction and maintenance of clinical remission in CD. CDED is a diet which excludes foods hypothesized to aggravate intestinal permeability, induce dysbiosis and colonic inflammation (Table 1). After induction of remission with 50% CDED and 50% EN, patients were instructed to consume 75% of their energy requirements through CDED and 25% through EN. Patients in the control group consumed a normal diet with 25% MEN. After six weeks, 28/32 (88%) of patients following CDED were still in clinical remission, compared to 14/25 (56%) in the control group (*p* < 0.05). Of note, FC levels reduced in the intervention but, this was not statistically significant (FC, μg/g, mean; CDED: baseline: 1744 vs. follow-up: 732, *p* = 0.22; control: baseline: 1021 vs. follow-up: 1589, *p* = 0.36). Although patients in the CDED group consumed only a small amount of MEN, it remains difficult to fully disentangle the extent to which the continued intake of MEN contributed to higher remission rates. Although changes in the composition of the gut microbiome were similar during the induction of remission between the two groups, there was a rebound in the relative abundance of Proteobacteria in the control group during the maintenance phase, compared to a sustained reduction in the CDED + MEN group. Clostridia continued to be higher in both groups, whereas there was a small rebound in Actinobacteria in both groups, compared to the beginning of treatment. Shannon diversity did not significantly change in any of the two groups.

##### UC Studies

Dairy-Free Diets

Lactose is a dietary component that has been implicated in UC, with dairy products being frequently excluded among patients [86]. Wright et al. [79] showed in a RCT that 1-year clinical relapse rates were not significantly different between patients with UC following a dairy-free diet compared to patients on a normal diet (dairy-free: 62% vs. normal: 78%, *p* = NS). Similarly, in another RCT, children with UC following a cow’s milk protein elimination diet had similar clinical relapse rates compared to children on a normal diet (intervention: 54% vs. control: 53%, *p* = 1) [81].

High-Fiber Diet

Contrary to the prevailing notion of the beneficial properties of fiber, the results from an uncontrolled trial in patients with UC showed that high fiber intake, particularly in the form of oat bran, may result in worse outcomes [80]. Sulfasalazine was the main maintenance therapy, with patients adhering to the high-fiber diet being advised to discontinue sulfasalazine two weeks after the start of the intervention. After a one-year follow-up, 75% of patients in the high-fiber group relapsed, compared to 20% in the normal diet group, suggesting exacerbation of disease activity due to high fiber consumption and/or discontinuation of sulfasalazine. Evidence from a systematic review on supplementation studies showed that fiber supplementation was not effective in maintaining clinical remission in CD [87]. However, supplementation with Plantago ovata, a fermentable fiber, was associated with lower relapse risk in patients with inactive UC, with the authors suggesting that this resulted from an increased production of butyrate [88]. This emphasizes that the role of fiber in IBD has not been fully understood and might differ between CD and UC.

Anti-Inflammatory Diet

Another exclusion diet assessed in an RCT in patients with UC, only available as an abstract, was the “Alberta-based anti-inflammatory diet” [82], which limited red meat and recommended consumption of pre- and probiotics, soluble fiber and ω-3 fatty acids. No significant differences were observed in the relapse rates between patients following the exclusion diet and patients in the control group following general dietary advice. However, the levels of FC decreased by almost 50% in the intervention group and underwent a threefold increase in the control.

Carrageenan-Free Diet

The effect of a carrageenan-free diet on relapse rates in patients with UC was assessed in a pilot, double-blind RCT [83]. Carrageenan is a widely used food additive, causing colonic inflammation in animal models of colitis [89]. Patients in both groups followed a carrageenan-free diet, with those in the intervention group consuming placebo, and those in the control group, carrageenan-containing capsules. Three patients dropped out in the intervention group. Relapse rates, assessed with disease activity indices or need for escalation of treatment, were significantly lower in the intervention than the control group, after one year, in per-protocol analysis (intervention: 0/7 [0%] vs. control: 3/6 [50%]), *p* = 0.046). FC levels did not significantly change in any group (FC, mg/g, mean ± SD; intervention: baseline: 149 ± 112 vs. follow-up: 111 ± 91, *p* = NS; control: baseline: 133 ± 125 vs. follow-up: 171 ± 143, *p* = 0.06).

##### Summary

Out of all the different food-based strategies explored in the present review, CDED + MEN, the semi-vegetarian diet and the carrageenan-free diet demonstrated signals of efficacy in maintaining clinical remission. However, these signals were mostly associated with maintenance of clinical disease activity and symptomatic improvement, rather than control of gut-specific inflammation. These presumptive dietary therapies should be further studied in larger, multicenter RCTs and their efficacy should be confirmed using objective colonic inflammation or endoscopic markers, before strong, clinical recommendations can be made. The common feature of these three diets is the restriction of food additives. However, in the CDED protocol, food additives are not completely eliminated since EN formulas are heavily industrialized and abundant in food additives (i.e., maltodextrin) [19]. Due to food industrialization, the exposure of patients to emulsifiers and other food additives has increased [90]. Animal studies and in vitro experiments have shown that exposure to carrageenan, maltodextrin, polysorbates and carboxymethyl cellulose may have detrimental effects on gut health [78]. However, the translation of findings from animal experiments to human CD is required before any dietary recommendations can be made. A recent analysis of 61 different EEN formulas, used for the induction of remission in CD, demonstrated no significant differences in remission rates between patients consuming EEN formulas containing at least one food additive implicated in IBD pathogenesis, compared with patients who consumed EEN formulas that did not contain those food additives, highlighting that the role of food additives in IBD has not yet been elucidated [19].

Current evidence regarding the rest of the food-based therapies assessed in the present review did not demonstrate their efficacy for maintenance of clinical remission in IBD. Although both the anti-IBD and the anti-inflammatory diet showed initial signals of efficacy, conclusions cannot be made unless the full peer-reviewed publications are available. Among other diets anecdotally reported in IBD, are diets low in fermentable oligosaccharides, disaccharides, monosaccharides and polyols (FODMAP), mostly recommended for the management of irritable bowel syndrome (IBS). Since IBS-like symptoms are regularly reported in IBD, patients might receive some symptom relief by following low-FODMAP diets [91,92]. However, there is no scientific evidence that these diets are effective for controlling colonic inflammation and maintaining clinical remission, neither in patients with CD nor UC.

### 2.3. Diet and Risk of Relapse: Epidemiological Evidence

Five prospective, observational studies (*n* = 2463 adults) investigating the association of diet with relapse risk were identified. Two trials had UC-only cohorts [93,94], one a CD-only cohort [95] and two studies recruited patients with both conditions [96,97]. Dietary assessment was performed only at baseline with the use of food frequency questionnaires [93,94,97], a dietary screener questionnaire [96] or a dietary history questionnaire [95].

Dietary temperance, assessed by a single question, about how much patients restricted their habitual diet (strong temperance: very frequent avoidance of foods) was associated against relapse risk in 76 patients with CD [95]. Moderate dietary temperance was negatively associated with time to relapse, suggesting that patients who occasionally consumed foods of their preference, took longer to relapse compared to those who rarely excluded foods. A high ratio of ω-6/ω-3 fatty acids was associated with a 46% lower risk of relapse. However, high CRP levels, the presence of past surgery and corticosteroid usage were also significantly positively associated with time to relapse, thereby potentially masking the true contribution of dietary factors on relapse risk.

The association of fiber intake and relapse risk was examined in two studies, which provided contradicting results [96,97]. Intakes of fiber and whole grain foods in the highest quartile were associated with 42% and 37% lower risk of relapse in 1130 patients with CD, compared to intakes in the lowest tertile, after multivariate adjustment [96]. No significant associations were observed in patients with UC. Conversely, in another study, high fiber intake, was positively associated with increased relapse risk, in 165 patients with IBD (OR [95%CI]: 3.65 [1.44–9.26]) [97]. The results were not adjusted for disease characteristics and were not reported separately for patients with CD and UC.

In a multi-center study, Barnes et al. [94] associated the intake of specific fatty acids, processed meat and alcohol with relapse rates. Patients with an intake of myristic acid in the third tertile, were more likely to relapse, compared to those on the first tertile, after multivariate adjustment (OR [95% CI]: 3.01 [1.17–7.74]). There were no significant associations between the consumption of processed meat and alcohol with the risk of relapse. Conversely, Jowett et al. [93] showed that patients with UC reporting a high intake (third tertile) of protein, meat and especially red meat, had increased risk of disease relapse compared to patients with a lower intake (first tertile), after multivariate adjustment (red and processed meat, OR [95%CI]): 5.19 [2.09–12.9]). The authors suggested that their findings could be attributed to sulfur, a dietary component, which has been associated with an endoscopically active disease phenotype [98].

#### Summary

In all studies, diet was assessed once at baseline, and therefore may not be representative of the diet at follow-up, when disease activity was assessed. Overall, the epidemiological evidence investigating the association of dietary components with disease recurrence is considerably limited and somewhat contradictive and inconclusive, highlighting the need for further, well-designed studies.

## 3. Future Directions

The compelling efficacy of EEN in ameliorating active disease in pediatric CD highlights the importance of diet in the management of IBD. There is remarkable ongoing research activity surrounding food-based treatments in active IBD, with the specific carbohydrate diet, the CD treatment with eating (CD-TREAT) diet, CDED and the autoimmune protocol diet, being some prime examples [78,99,100,101]. CD-TREAT, a novel food-based diet, designed to replicate the nutritional composition of EEN, has shown promising clinical efficacy in a pilot trial in patients with active CD [99]. The prevailing paradigm regarding the mechanism of action of CD-TREAT is similar to that of EEN, since the modulation of the gut microbiome has been linked to both dietary therapies [5,99].

Although the scope of our review focuses on food-based dietary therapies, it is worth mentioning that there is a plethora of various dietary components that are not discussed in detail here, and which could play a role in controlling disease activity or gut symptoms in patients with IBD in remission [102]. Indicative examples include single-nutrient supplements, such as vitamin D, ω-3 fatty acids and zinc, nutritional components, such as flavonoids, bioactive compounds (e.g., curcumin), prebiotics and other non-nutritional compounds, such as probiotics and short chain fatty acids [103]. More information about these dietary components can be found in comprehensive, recently published reviews [102,103].

Although MEN consumption seems a promising strategy for disease maintenance in CD, long-term adherence may be challenging for some patients [104]. Hence, there is an unmet need to identify the exact dietary components triggering disease activity and develop sustainable, long-term dietary strategies based on the exclusion of these components. We propose several essential and desirable features that we believe should be incorporated in the design of trials investigating dietary therapies for maintenance of clinical remission and observational studies, aiming to identify dietary triggers of relapse in IBD (Table 2).

Trials investigating strategies for maintenance of clinical remission should have an RCT design, be adequately powered, and ideally involve multiple recruitment centers (Table 2). Pilot, uncontrolled studies are important to test early efficacy signals and the feasibility of an intervention. Cohort studies should employ a prospective design to minimize the potential bias of reverse causation (Table 2). Long-term follow-up, which would allow an adequate number of endpoints and a representative sample size are essential characteristics. Since CD and UC have different disease phenotypes, both intervention and cohort studies should include homogeneous populations. When not feasible, stratification according to disease phenotype is required during statistical analysis.

Outcomes of remission and relapse should be defined using a combination of routinely applied clinical disease activity indices and objective biomarkers of systemic (e.g., ESR, CRP) and intestinal inflammation (e.g., FC). Once more, the difference between symptom response and objective markers of luminal disease activity is important in distinguishing between nutritional strategies aimed at prevention of symptom recurrence versus avoidance of relapse. While, this may seem cumbersome to labor the point, clarifying this distinction as objectively as possible, will be essential in moving nutritional research forward in IBD.

As patients will most likely be on concomitant maintenance treatment with other drugs at the time of recruitment, stratification based on maintenance medication is recommended. Adjustment for covariates, which might influence the outcome of interest should be performed during statistical analysis to minimize bias in exploring associations between dietary components and risk of relapse. For studies evaluating food reintroduction protocols, we recommend, if possible, the collection of multiple, serial fecal samples (recognizing the practical issues around this). This will help to best associate dynamic changes in the gut microbiome, with short-term changes in dietary intake [105].

The assessment of dietary compliance and overall dietary habits holds pivotal importance and should be performed in every study. Dietary assessment should be performed both, at baseline and at the end of the follow-up period and ideally multiple times throughout the observational period. This would assess the uniformity of the dietary pattern throughout the study period and provide a better representation of the association between diet and the disease outcome. New technologies, such as web-based applications for dietary assessment may be incorporated. However, these must first be validated within the target populations. Up-to-date food composition tables incorporating complete dietary information should be used and the intake of food groups, food additives and dietary patterns should be analyzed, in addition to that of nutrients. As new molecular-based techniques are becoming available, the use of blood or fecal metabolites as food biomarkers could be incorporated as a complementary method to conventional dietary assessment.

To our knowledge, among the included studies using food-based therapies for maintenance of remission in IBD, the assessment of the gut microbiome was only performed in the trials by Levine et al. [78] and Mutlu et al. [76]. This highlights a gap in the characterization of the mechanism of action in studies assessing food-based therapies. With the rapid advance of high-throughput profiling, examination of the gut microbiome and the immune system has now become more feasible. Therefore, it should be an integral part of every dietary study in IBD, aiming to understand underlying mechanisms by which diet provokes inflammatory response and the mediating role of the gut microbiome [106]. Along with novel nutritional therapies, microbial therapeutics could also serve as a potential paradigm-changing strategy for the management of IBD, which could complement the established medical treatment and form a triad, to improve disease management and quality of life of patients with IBD.

## 4. Conclusions

There are compelling reasons to believe that specific dietary triggers of gut inflammation in IBD exist and can be identified. Therefore, an urgent need exists to design high-quality dietary studies, in order to both mobilize diet as a therapy, and simultaneously unravel these triggers and their mechanisms of action in driving disease pathology. Such an approach would likely lead to reduced treatment costs and provide new insights into the disease itself. The onset of multiple ’omics technologies and computational tools in recent years, means we are now capable to advance our knowledge in the area of nutritional research even further, understand the complex interplay between diet, the host and the gut microbiome and develop personalized dietary treatment options to control disease activity.

## Figures and Tables

**Table 1:** Summary of food reintroduction protocols and food-based dietary therapies which have been used as a strategy for maintenance of clinical remission in IBD in studies which included a control group.

Dietary Regime	Reference, Setting	Cohort	Included Dietary Components	Excluded Dietary Components	Control Group	Clinical Efficacy (Disease Activity)	Clinical Efficacy (FC/Endoscopy)
**Food reintroduction protocols in CD**							
Symptom-alleviating food reintroduction diets	Jones et al., 1985 [41], UK	CD	Personalized diet: single, daily food reintroduction	Symptom-triggering foods (most common: wheat, dairy, vegetables)	Unrestricted diet	✓	NR
	Riordan et al., 1993 [46], UK	CD	Personalized diet: single, daily food reintroduction	Symptom-triggering foods (most common: corn, wheat, milk, yeast)	Unrestricted diet + corticosteroids	✓	NR
LOFFLEX diet	Woolner et al., 1998 [44], UK	CD	Low-fat (~50 g/d) + low-fiber (~10 g/d) diet: lean meat, fish, soy milk, rice, olive oil, ≤2 portions of vegetables & fruit/day, refined sugars	Red meat, processed fish, dairy, most grains, pulses, >2 dried portions of fruit and vegetables/day, tea, coffee, nuts, alcohol, sauces	Gradual food reintroduction	✕	NR
Rapid food reintroduction	Faiman et al., 2014 [47], UK	CD	Unrestricted diet following initial 3-day low-residue diet post EEN completion	High-residue foods for 3 days post EEN completion	Gradual food reintroduction	✕	NR
IgG exclusion diet	Wang et al., 2017 [45], China	CD	Personalized diet (foods not causing increased IgG levels and symptoms)	Foods causing high IgG levels (most common: rice, tomato, egg, maize)	Unrestricted diet	✕	✕
**Food-based therapies in IBD**							
Low-refined carbohydrate diet	Ritchie et al., 1987 [73], UK	CD	Low-refined, fiber-rich diet, consumption of only unrefined carbohydrates	Foods containing white flour and sugar	Low-fiber, no restriction in refined carbohydrates	✕	NR
Low-carbohydrate diet	Lorenz-Meyer et al., 1996 [74], Germany	CD	Carbohydrates <84 g/d, specific foods not reported	NR	Habitual diet	✕	NR
Semi-vegetarian diet	Chiba et al., 2010 [75], Japan	CD	Daily: vegetables, fruit, miso, rice, legumes, yoghurt; weekly: fish; fortnightly: meat	Cheese, bread, sweets, fast food, juices, carbonated drinks	Omnivorous diet	✓	NR
Anti-IBD diet *	Mutlu et al., 2016 [76], USA	CD	NR	Wheat and most other grains, animal fat, additives, preservatives	Habitual diet + FOS supplement Habitual diet + placebo	✓ ✕	NR NR
Low red & processed meat diet	Albenberg et al., 2019 [77], USA	CD	All except red & processed meat	Red & processed meat (≤1 servings/month)	≥2 servings of red & processed meat per week	✕	✕
CDED + 25% MEN	Levine et al., 2019 [78], Israel, Canada	CD	MEN: 25% of energy requirements mandatory: chicken, eggs, potatoes, banana, apple; recommended: fruit & vegetables	Gluten, dairy products, red and processed meat, food additives, coffee, alcohol	Normal diet + 25% EN	✓	✕
Dairy-free diet	Wright et al., 1965 [79], UK	UC	All except dairy products (butter allowed)	All dairy products including cheese	Exclusion of fried foods, condiments	✕	NR
High-fiber diet	Davies and Rhodes 1978 [80], UK	UC	Oat bran supplement (25 g/d), increased intake of wholewheat cereals, vegetables	NR	Habitual diet	✕	NR
Cow’s milk protein elimination diet	Strisciuglio et al., 2013 [81], Italy	UC	All except dairy products	All dairy products including cheese and butter	Habitual diet	✕	NR
Alberta-based anti-inflammatory diet *	Keshteli et al., 2016 [82], USA	UC	Increased intake of prebiotics, soluble fiber, ω-3 fatty acids	Red and processed meat	Normal diet	✕	**?**
Carrageenan-free diet	Bhattacharyya et al., 2017 [83], USA	UC	All except carrageenan-containing products	Products containing carrageenan (e.g., yogurt, ice cream, processed meat, beer)	Same diet + carrageenan-containing capsules	✓	✕

**✓**: Higher clinical efficacy signals, defined as significant improvement in disease activity indices, FC levels or endoscopic indices in the intervention diet group, compared to the control group; **✕**: Equal/lower clinical efficacy signals in intervention diet group, compared to the control group; **?**: Unclear evidence; NR: not reported; * only presented as an abstract. CD: Crohn’s disease, CDED: Crohn’s disease exclusion diet, EN: Enteral nutrition, FC: Fecal calprotectin, FOS: fructooligosaccharides, IgG: Immunoglobulin G, IBD: Inflammatory bowel diseases, MEN: Maintenance enteral nutrition, UC: Ulcerative colitis.

**Table 2:** Essential and desirable characteristics of intervention trials aiming to assess the efficacy of dietary strategies for the maintenance of clinical remission and cohort studies aiming to investigate the association of dietary components with the risk of relapse in patients with IBD.

Essential Characteristics	Trials	Cohort Studies
Randomized controlled design	✓	n/a
Prospective cohort design	n/a	✓
Large, representative sample	✓	✓
Long-term follow-up suitable for identification of adequate number of clinical relapses	✓	✓
Separate analysis for UC and CD cohorts	✓	✓
Definition of remission and relapse outcomes using disease activity indices and objective biomarkers of systemic (e.g., CRP, ESR) and intestinal (e.g., FC) inflammation	✓	✓
Adjustment for use of maintenance drugs and other covariates	✓	✓
Dietary assessment	✓	✓
Analysis of nutrients, food groups and food patterns	✓	✓
Use of up-to-date, complete food composition tables for dietary analysis	✓	✓
Assessment of mechanism underlying the mode of action of diet (e.g., metabolome, microbiome, immune profile)	✓	✓
**Desirable characteristics**		
Multicenter design	✓	✓
Homogeneous population (only CD/only UC)	✓	✓
Endoscopic assessment	✓	✓
Use of novel technologies for dietary assessment (e.g., web-based tools)	✓	✓
Analysis of other dietary components (e.g., gluten) and non-nutrient components (e.g., food additives)	✓	✓
Incorporation of food biomarkers in dietary assessment (e.g., plasma/fecal metabolites)	✓	✓
Collection of serial fecal samples during early food reintroduction to assess dynamic changes in gut microbiome	✓	✓
Dietary assessment at multiple timepoints throughout the study to assess uniformity of dietary habits	✓	✓

**✓**: Higher clinical efficacy signals, defined as significant improvement in disease activity indices, FC levels or endoscopic indices in the intervention diet group, compared to the control group; CD: Crohn’s disease, CRP: C-reactive protein, ESR: Erythrocyte sedimentation rate, FC: Fecal calprotectin, n/a: not applicable, UC: Ulcerative colitis.

## Supplementary Materials

The following are available online at https://www.mdpi.com/2072-6643/12/7/2018/s1, File S1: Summary of studies investigating the role of diet for maintenance of clinical remission in IBD.
